# Evaluation of depression, stress and quality of life indexes in patients with atopic dermatitis^☆^^☆☆^

**DOI:** 10.1016/j.abd.2020.09.010

**Published:** 2021-07-17

**Authors:** Cleide Rodrigues de Castro, Maria Elisa Bertocco Andrade, Renata Marli Gonçalves Pires, Mario Cezar Pires

**Affiliations:** a Complexo Hospitalar Padre Bento de Guarulhos, Guarulhos, SP, Brazil; b Faculty of Medicine, Universidade de São Paulo, São Paulo, SP, Brazil

Dear Editor,

Atopic Dermatitis (AD) is a recurrent chronic inflammatory dermatosis, characterized by pruritus, sleep disorders and multiple comorbidities, with significant psychological alterations and impact on mental health.^1^ In many cases, there is loss of quality of life, with deep psychosocial and economic impact.^2^

Some authors have described different degrees of depression and stress in patients with AD.^1^, ^3^ In this study, the authors evaluated depression, stress and quality of life indexes in adults with AD using:

• Beck depression inventory (Beck-BDI-II depression inventory - validated as of 15 years old)^4^;
• Inventory of stress symptoms for adults (ISSL - validated as of 15 years old) Classifies into 4 phases: alert (contact with aggressive stimulus); resistance (adaptation of the body); near exhaustion (depletion) and exhaustion (physical and/or psychological impairment)^5^;
• Dermatology life quality index (DLQI-BRA).

The analyzed variables were: age; gender; the visual scale of daytime and nighttime pruritus, the severity of AD (SCORAD - atopic dermatitis score) using Pearson's correlation with indexes of depression, stress and quality of life.

Statistical analysis was performed using the Adans Statistics Company (CONRE 8250-A).

Thirty-one patients were included, of which 18 were women, 13 men, with a mean age of 29 years, and a standard deviation of 12.2 (minimum 15; maximum 54 years). Of the 31 patients, 7 (22.6%) had mild depressive symptoms, while 5 (16.1%) had mild to moderate; 18 (38.7%) had moderate to severe and 7 (22.6%) had severe symptoms (Fig. 1). Sleep pattern disorders had the highest score (1.74), followed by indecision (1.58), low self-esteem (1.42), and self-criticism (1.39). Suicidal thoughts or ideation had the lowest score (0.26), followed by pessimism (0.55) and loss of sexual interest (0.55).

**Figure 1:**
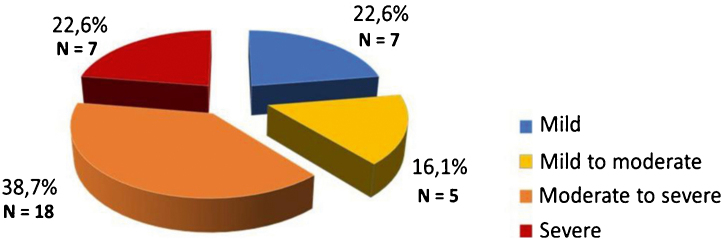
Beck II depression inventory.

Of the 30 patients with symptoms of physical stress, 18 (60%) were in the resistance phase, while 11 (36.7%) were in the near-exhaustion phase and only 1 (3.3%) was in the alert phase (Fig. 2). Symptoms of psychological stress were manifested by 22 patients (73.3%), while 4 (13.3%) also reported physical symptoms and another 4 (13.3%) manifested both. There was no association between stress, depression and impaired quality of life with gender or age.

**Figure 2:**
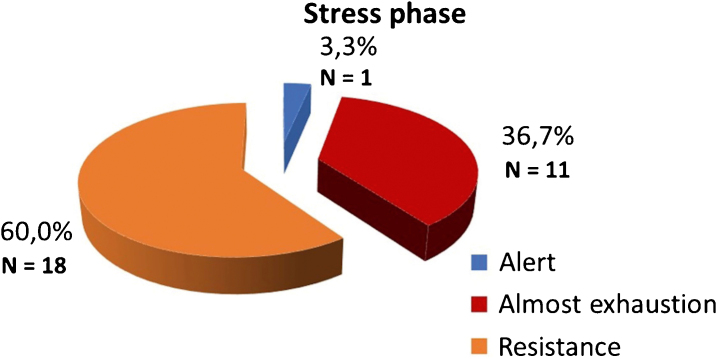
Stress phases.

The DLQI-BRA classification showed patients with significant quality of life impairment as follows: 7 (22.6%) showing mild impact, 6 (19.4%) moderate, 14 (45.2%) severe and 4 (12.9%) very severe impact (Fig. 3). The correlation coefficient between daytime pruritus and physical stress was 0.385; with psychological stress, 0.369; with both, 0.412; with depression, 0.311; with quality of life, 0.127. A moderate correlation was observed with stress and depression, and a weak correlation with quality of life. The correlation between depression and anxiety was 0.608, considered moderate to strong.

**Figure 3:**
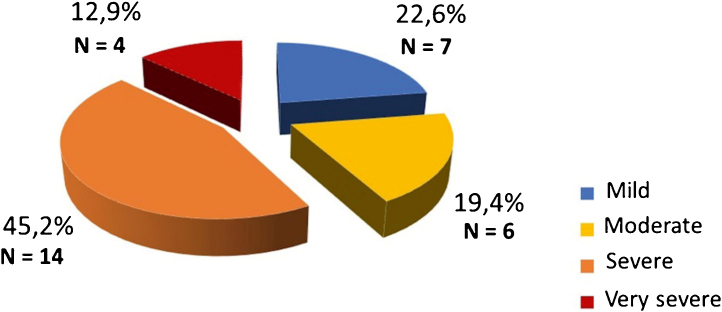
Dermatology Life Quality Index (DLQI) Questionnaire.

The correlation coefficients of SCORAD with physical/psychological stress and depression were non-significant (-0.02 and -0.06 respectively, p-value 0.911 and 0.739). There was a weak positive correlation between the degree of severity and impaired quality of life (0.268, p-value = 0.145), which is in accordance with Carvalho et al.^2^ Because it is a reference for the health network for dermatological diseases, most patients had moderate to severe disease; thus, it was concluded that the stress, depression and impaired quality of life indexes do not depend on AD severity.

Psychosocial and emotional factors affect the course of the disease, as well as the response to therapeutic interventions. Other authors have obtained similar indexes of depression (around 15% for severe cases).^1^, ^3^ Silverberg et al. stated that dermatologists should consider these associations when evaluating AD patients.^1^ Unlike other authors, the present study found no association with suicidal thoughts or ideation (0.26).^3^

The present study found a high prevalence of stress (96.8% of participants), with the majority (60.0%) in the resistance phase and a significant predominance of psychological symptoms (73.3%), which was more relevant than in other studies.^1^, ^3^

The most common psychological symptoms were: sudden increase in motivation; enthusiasm; insecurity; feeling of incompetence; constant thinking on a single subject; excessive irritability; nightmares and fatigue. Classically, these findings are observed when living with greater vulnerability to psychological stress, as it occurs in AD crises. On the other hand, in our experience with atopic dermatitis patients we observe anticipatory distress and self demand, which in turn can lead to crises, creating a vicious circle (personal opinion of the authors).

The authors conclude that individuals affected with atopic dermatitis often show symptoms of depression, stress and impaired quality of life. Itching is among the symptoms that were most often related to psychological distress and reduced quality of life. Thus, it is recommended that dermatologists, when treating patients with AD, also analyze aspects such as stress, depression and quality of life.

## Financial support

None declared.

## Authors’ contributions

Cleide Rodrigues de Castro: Design and planning of the study; drafting and editing of the manuscript; collection, analysis, and interpretation of data; critical review of the literature; critical review of the manuscript.

Maria Elisa Bertocco Andrade: Intellectual participation in the propaedeutic and/or therapeutic conduct of the studied cases.

Renata Marli Gonçalves Pires: Critical review of the literature; critical review of the manuscript.

Mario Cezar Pires: Approval of the final version of the manuscript; design and planning of the study; effective participation in research orientation; intellectual participation in the propaedeutic and/or therapeutic conduct of the studied cases; critical review of the literature; critical review of the manuscript.

## Conflicts of interest

None declared.
